# Do we actually need aging clocks?

**DOI:** 10.1038/s41514-025-00312-2

**Published:** 2025-12-19

**Authors:** Dmitrii Kriukov, Evgeniy Efimov, Mikhail S. Gelfand, Alexey Moskalev, Ekaterina E. Khrameeva

**Affiliations:** 1 Skolkovo Institute of Science and Technology, Moscow, Russia; 2 Artificial Intelligence Research Institute, Moscow, Russia; 3 Institute of Longevity, Petrovsky Russian Research Center for Surgery, Moscow, Russia

**Keywords:** Biomarkers, Epigenetics, Ageing

## Abstract

Aging clocks use machine learning to estimate biological age as a proxy for general health state. Here, we critically examine their practical value, highlighting fundamental challenges: abstract definitions, inconsistent clinical validation, and ignored prediction uncertainty. By comparing aging clocks with expert risk scores, direct outcome predictors, and emerging large health models, we question their benefits and encourage researchers to explicitly justify clock advantage over established alternatives, ensuring truly actionable insights.

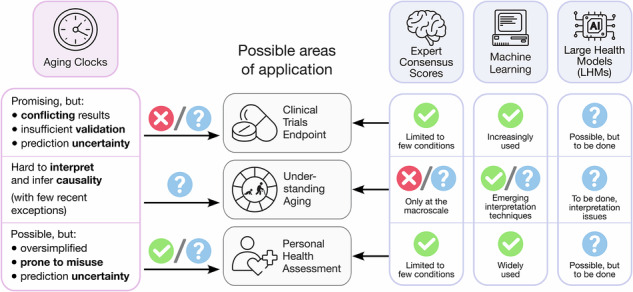

## The rise and promise of biological aging clocks

Aging clocks are defined as computational algorithms intended to estimate an individual’s biological age (as opposed to chronological age), which increasingly capture the attention of both researchers and practitioners in the fields of longevity science and medicine^1–3^ (Box 1). This surge of interest has been fueled by the growing availability of large-scale and high-throughput profiling of omics and other biomarkers associated with aging^4,5^. Consequently, a plethora of studies has emerged, producing numerous aging clock models trained using different and sometimes rather unconventional biomarkers: DNA methylation^6,7^, plasma proteins^8,9^, urine metabolites^10^, clinical blood tests^11^, facial images^12^, X-ray scans^13^, and many others^14^.

The interest in estimating biological age is driven primarily by the desire to achieve long-standing goals shared by longevity researchers and clinicians alike. *First*, biological age may serve as a surrogate endpoint in clinical trials of geroprotective interventions (that is, drugs or other therapies presumed to extend lifespan or reduce the burden of chronic age-related diseases). This would provide a fast and affordable way to measure treatment effects, not requiring observations that span decades to track actual lifespan changes. *Second*, by analyzing how biological age estimators work, we could better understand the fundamental processes that drive aging. *Third*, biological age could represent the overall health status of an individual with a single measure in a succinct and rapid way. Importantly, when derived from a specific data type, the biological age can serve either as a context-specific surrogate biomarker (e.g., epigenetic, proteomic, clinical blood-based, etc.)—used in conjunction with other biomarkers—or as the output of a higher-level integration procedure that combines multiple “biological ages” into a generalized estimate of overall health status.

However, the key question remains unanswered: do we actually need the abstract concept of biological age to achieve these goals? And if so, then how exactly should we employ it? By summarizing the latest advances and examining current challenges in aging clock research, our Perspective strives to address these critical questions.

Box 1 Definitions of terms used in this work **Biological age**: conceptually, an individual’s age that corresponds to the chronological age at which the average person in a reference population shares the individual’s pattern of age-dependent biological features. In practice, it is intended to satisfy four criteria: (a) it allows to predict remaining lifespan better than does chronological age; (b) it allows to predict the time-to-onset of chronic age-related diseases better than does chronological age; (c) it allows to distinguish patients with age-related diseases from healthy individuals of the same chronological age; (d) it is expressed in time units^2,3^.

**Aging clocks**: computational algorithms designed to estimate an individual’s biological age.

**Direct outcome prediction**: prediction of measurable health outcomes (e.g., onset of age-related diseases, morbidity, functional decline, or mortality) directly from features, without using biological age as an intermediate target.

**Domain of applicability**: the empirical data domain (species/tissue/assay/age range) on which a model was trained and validated; applying the model beyond this domain is considered as an out-of-domain (OOD) prediction, which is coupled with a higher uncertainty of that prediction.

**Large Health Model (LHM)**: a computational model—inspired by large language models (LLMs)—that represents human health as a longitudinal sequence of clinical and health-related events. Trained on massive, high-quality longitudinal datasets, LHMs learn the temporal dynamics and conditional dependencies among health events (e.g., diseases, interventions, biomarkers), enactcomes such as mortality or multimorbidity.

## Hard to define, even harder to validate

Biological age represents an individual’s overall health state—an abstract entity meant to reflect a fundamental property of organisms that cannot be directly observed or quantified in nature, making it tremendously difficult to define. It has been referred to as a health index^15^, a surrogate biomarker of aging^16^, or a latent variable describing the integrative physiological state of an organism^17^. However, these descriptions can hardly translate into actionable definitions for constructing an estimator of biological age. Consequently, being abstract and non-measurable, biological age exists only as the output of the algorithm claiming to measure it; therefore, it is defined by the specific training data and underlying model architecture^18^. Essentially, every new clock defines its own biological age.

Formally, biological age is defined as a numerical value designed to satisfy certain essential criteria^2,3^, which can be viewed as a proposed standard for aging clocks development: (a) biological age should predict the remaining lifespan better than does the chronological age (time elapsed since birth) of an individual; (b) it should also predict the time-to-onset of various chronic age-related diseases better than does chronological age; (c) it should be able to distinguish patients with age-related diseases from healthy individuals of the same chronological age; and (d) for practical convenience, it should be expressed in the same time units as chronological age. A measure that fulfills all these properties is difficult to construct, but it would undoubtedly have enormous practical value. In addition, aging clocks should demonstrate high reproducibility in independent datasets and responsiveness to interventions. Furthermore, explainability and generalizability (e.g., across tissues or populations) would also be highly desirable.

We acknowledge that no single existing clock can be expected to capture all manifestations of aging and disease. There can even exist disease-specific “clocks”^19^ which can hardly be called aging clocks per se, but could serve as disease predictors in their own right. However, a useful predictor of biological age should consistently reflect systemic aging processes, showing broad associations with multiple age-related diseases, multimorbidity, and mortality, rather than with isolated or age-independent conditions. In practice, the usefulness of a given clock depends strongly on the research question and context; before applying any existing model, investigators should first determine whether a biological age estimate is necessary for their specific aim using systematic meta-analyses of clock performance in the respective contexts, and also understand what additional insight (beyond established biomarkers) the clock’s use can provide.

Furthermore, how accurately do the existing biological age estimators (also referred to as predictors) allow us to predict the outcomes of clinical trials (goal 1), or generate insights about the fundamental mechanisms of aging (goal 2), or quickly assess the overall health state of an organism (goal 3)? And how well do they satisfy the four formal criteria? In our previous work^3^, we have introduced an open-source benchmarking platform for testing the epigenetic aging clocks’ ability to satisfy this standard. In a separate study, we have demonstrated why clocks should be constructed using algorithms that quantify their own uncertainty and have provided a working example^20^.

**Fig. 1:**
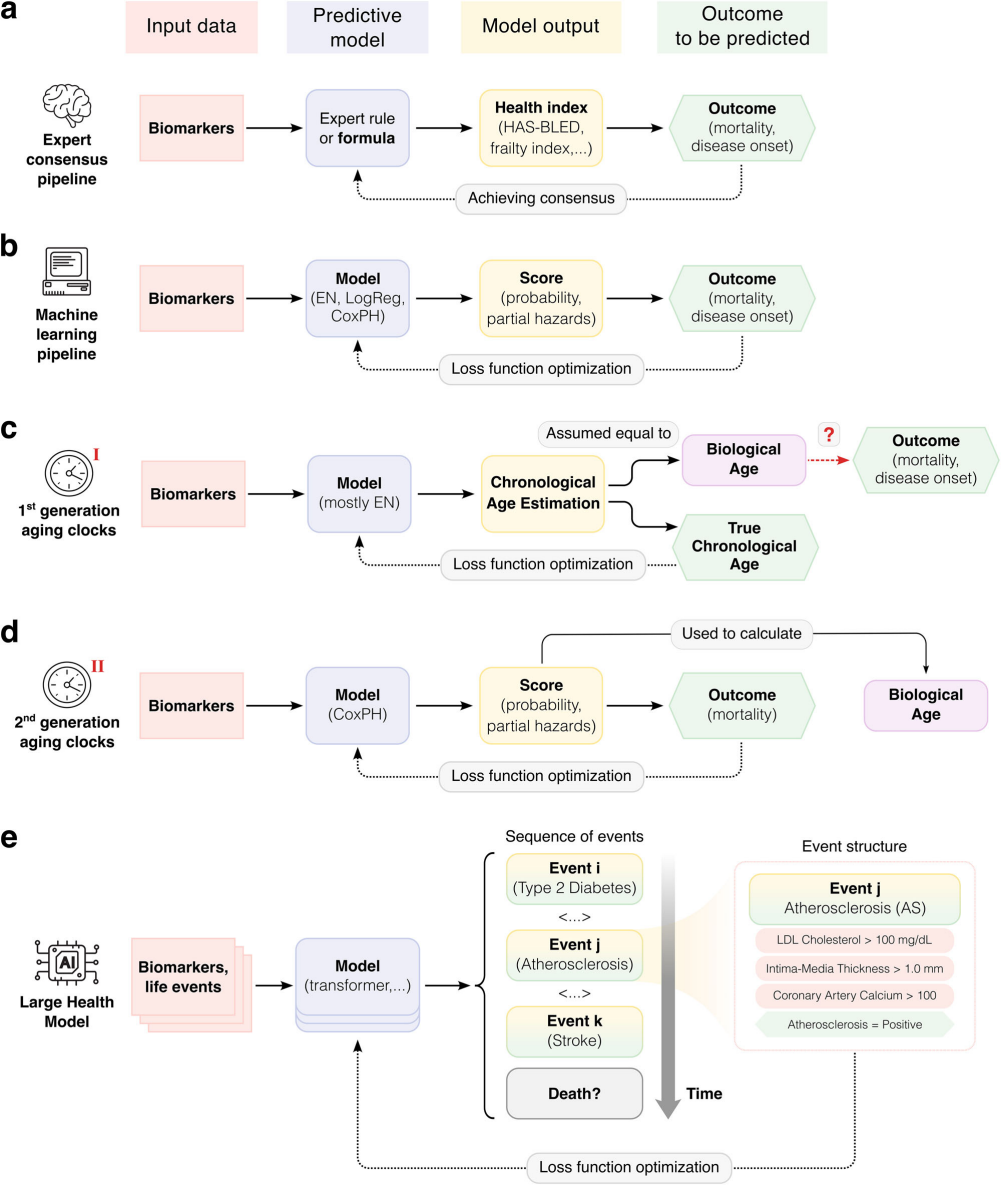
Approaches to predicting health outcomes. **a** Classical expert consensus-based pipeline. **b** Machine learning (ML)-based pipeline. **c**, **d** Two generations of aging clocks primarily focus on inferring biological age, which is then used to predict health outcomes. Notably, the training procedure of first-generation clocks is typically detached from predicting the actual health outcomes. **e** Emerging large health models (LHMs) directly predict sequences of events (biomarker values or health outcomes) instead of univariate scores. EN - elastic net penalized linear model, LogReg - logistic regression model, CoxPH - Cox proportional hazard model, LDL low-density lipoprotein.

In most studies, however, researchers validate the aging clocks they propose against only a few of these requirements. For instance, they often test whether an aging clock assigns significantly increased ages to patients with Hutchinson-Gilford progeria syndrome^21^, cancers^5,22^, type 2 diabetes^23^, etc. Other researchers evaluate whether an increase in the predicted age corresponds to higher hazard ratios in mortality risk predictions^6^. In essence, biological age is frequently used as a proxy for certain health outcomes (e.g., onset of age-related diseases, morbidity, functional decline, or mortality) that, while being directly measurable, are often costly to track. Thus, it becomes crucial to compare biological age predictors with alternative approaches that aim to address the same underlying questions that the aging clocks are designed to answer (Fig. 1).

## Four approaches to predicting clinical outcomes

Developing a surrogate biomarker that correlates with a clinical trial outcome (also referred to as an endpoint) and can partially replace it has long been sought for in many areas of biomedical research^24^.

Historically, the first approach to designing such a biomarker required reaching a consensus within an expert community regarding which primary biomarkers are the strongest risk factors (or predictors) of a given health outcome (Fig. 1a). Expert consensus is especially important when data from large cohort studies are lacking, but experts already recognize the significance of certain factors for inclusion in the scoring system. In gerontology, one such proxy biomarker is the frailty index^25^, which assesses vulnerability to adverse outcomes in senior individuals based on several simple factors. From a geroscience perspective, such composites operationalize the hypothesis that intervening in the core aging mechanisms may concurrently delay diverse morbidities and frailty^26,27^. Other examples include: intrinsic capacity^28^ (IC) score measuring an older adult’s composite functional ability, the ASA Physical Status Classification System used by anesthesiologists for assessing patient health before surgery^29^, the HAS-BLED score to predict the 1-year risk of major bleeding in patients with atrial fibrillation (AF)^30^, and the CHADS_2_ score for assessing stroke risk in patients with AF^31^. More advanced calculators—such as the ASCVD Risk Estimator^32^, SCORE2^33^, and PREVENT^34^, which are widely employed to estimate cardiovascular risks,—rely on statistical relationships to derive their expert formulas, instead of coefficients defined purely by expert consensus.

The advantage of this expert-based pipeline is its transparency: an interpretable set of primary biomarkers and their significance for predicting the target outcome are explicitly defined. The downsides are that experts may overlook hidden relationships between primary biomarkers and may have biased opinions regarding the importance of particular biomarkers (less relevant for model weighted expert formulas). Expert-based scores perform best when clinical consensus exists, which is currently lacking in aging biology. Because aging manifests as progressive, multi-system loss of function, a pragmatic alternative is to assemble a higher-level composite (like IC) from established, domain-specific markers (cardiac, pulmonary, cognitive, musculoskeletal, renal, sensory) and their validated, consensus-based scores. Although IC-style composites still require domain selection, weighting, and robust longitudinal and cross-population validation (and may miss very early molecular changes), they might offer a clearer path to consensus-based measurement and intervention than a single, monolithic biological age predictor. Recent studies also expose deeper structural limits: consensus methods like Delphi may achieve agreement without ensuring predictive validity or generalizability, making the authors explicitly call for rigorous prospective validation^35^; and a global survey reveals persistent lack of conceptual agreement on defining aging and selecting its key mechanisms, underscoring the fragility of expert-based methods^36^. To address some of these problems, and with the emergence of advanced data-driven statistical learning algorithms, the expert-based approach naturally evolved into a new approach that now rightfully forms a separate group.

The second approach is to build statistical, machine learning (ML) or, more recently, artificial intelligence (AI) models that directly predict health outcomes (i.e., predict them as not a result of training a separate model on biological ages but predict outcomes directly from features, without using biological age as an intermediate target) (Fig. 1b). These models are trained using a mathematically rigorous and explicit objective error function (also referred to as loss function) to minimize errors and maximize prediction accuracy. They have successfully been applied to predict mortality among cancer patients^37^ or in intensive care units^38^, and for many other clinical outcomes^39,40^. Moreover, ML-based scores, signatures, and other kinds of composite indices are being increasingly approved and adopted as surrogate endpoints in various clinical trials^41–43^. An inherent advantage of ML models is their ability to impartially evaluate the contribution of each factor to predictive performance without hand-tuned weights. However, this ability is only as good as the data—any biases, confounders, or measurement artifacts in the training set will be learned (and potentially amplified) by the model. Therefore, performance-based factors’ contributions must not be interpreted as causal effects. The major disadvantage of ML models is that interpreting the resulting model coefficients remains challenging. Although this issue has been addressed in recent years through explainable AI methods^44^, we still cannot be certain about the causality of the identified relationships with health outcomes.

The third approach is aging clocks, which employ ML model training pipelines to estimate biological age, and then use it as a surrogate biomarker to predict health outcomes (Fig. 1c, d). Traditionally, the performance of first-generation aging clocks is measured primarily by how accurately they predict chronological age. While a certain degree of correlation between the predicted and chronological ages is desirable, an absolutely precise clock would be helpful in forensics, but absolutely irrelevant for health assessment—a phenomenon called *the* *biomarker paradox*^3,17,45^. First-generation aging clocks (Fig. 1c) are trained solely to predict chronological age and are therefore highly susceptible to this paradox. Second-generation clocks (Fig. 1d) are methodologically more solid because they are trained to predict all-cause mortality, a perfectly measurable metric, while outputting biological age values as a by-product, for convenient comparisons with other clocks. In general, both clock generations (and especially the first one) require further external validation—for example, testing the associations between the predicted age and mortality or multimorbidity^8,46,47^. However, for all models, including epigenetic, other omics-based clocks, and non-clock ML algorithms, opportunities for such validation remain limited by data privacy constraints. Even when model weights are openly available, independent testing is possible only for few researchers with data access, and broader external validation would still benefit from a wider access to diverse datasets. To circumvent this, we have recently proposed an alternative, fully open-access strategy: testing aging clocks based on their ability to distinguish healthy individuals from those with conditions that accelerate aging^3^. We also note that some algorithms, such as DunedinPACE^48^, estimate the pace of aging (i.e., the average rate of biological age change per chronological year) rather than biological age per se. Our definition is intentionally limited to models predicting biological age to encompass the most common types of aging clocks. However, we note that DunedinPACE is a valuable biomarker that should undergo standardized validation like any other biomarker of aging.

To be included in clinical trials, any diagnostic tool must be thoroughly validated and exhibit stable predictions. Some ongoing trials include aging clocks as primary or secondary endpoints (among other biomarkers), but their results are yet to be seen^2^. From what we do already know based on the post hoc and exploratory analyses in the finished trials, aging clocks do not appear as stable, reliable endpoints: different models predict different age acceleration or lack thereof in different trials. For example, the CALERIE trial on long-term caloric restriction^49^ showed “significant reduction of DunedinPACE and PhenoAge (blood chemistry), but no significant effects for other biomarkers of aging” (GrimAge, Horvath and Hannum clocks), even though both PhenoAge and GrimAge are the second generation clocks trained on similar data types and could be expected to behave similarly for such long-term interventions, and the Horvath clock yielded accelerated aging in obesity^50^. The inconsistencies in clock predictions between biological and technical replicates have been demonstrated even stronger in a recent pre-print^51^. Thus, these and other examples from ref. ^2^ suggest that it might be too early to draw any solid conclusions from such conflicting predictions. Moreover, these cases can be viewed as studying the tool (clocks) using clinical trials, instead of gaining insights about clinical trials using the tool. Which is itself a possible venue of research, but it does not yet help us achieve the goal of estimating the effect of longevity interventions in a reliable way.

Hence, when we rely on aging clocks for health estimation, we obtain a measure that is challenging to interpret and validate. Why introduce an additional latent variable between an ML model and a health outcome if we can directly predict the latter using the former? It could be a perfectly rational strategy if the goal of first-generation clocks were only to predict the chronological age itself, as in forensic applications. However, once we focus on reliably predicting age-related health outcomes, the value of using an intermediate proxy biomarker becomes questionable. Moreover, by compressing all biomarker information into a single latent variable, we might weaken the ML model’s ability to accurately predict the outcome. This is analogous to using ML for inferring other implicit (latent) concepts in science and culture, such as levels of intelligence, consciousness, happiness, or even love. Although it can be done, how confidently can we trust machine learning algorithms to predict these abstract concepts from basic features? In summary, aging clocks seem to be less practical for health estimation compared to the first two classical approaches, while raising new challenges and paradoxes ^3,17,52^.

The fourth approach, by contrast, promises to notably advance the practical longevity research (Fig. 1e). Reliable prediction of mortality or (multi-)morbidity requires biomarkers data of unprecedented size, depth, and quality. In this context, it becomes impossible to ignore the elephant crawling into the room: artificial intelligence. It has been advancing exponentially over the past few years and can now operate with massive volumes of longitudinal (sequential) data, enabling a more comprehensive assessment of human health by directly predicting future life events (Fig. 1e). This approach is best exemplified by the so-called “large health models” (LHMs)^53–55^, which are based on the well-established methodologies for training large language models (LLMs). LHMs represent human health as a sequence of events (Fig. 1e) allowing us to identify which dysregulation events occur first, and to analyze how the conditional probability of one event (e.g., atherosclerosis) affects the occurrence of another (e.g., stroke). By uncovering these complex pathways of health-related events, we can gain a more nuanced, albeit observational, understanding of how human health evolves over time.

It is important to note that LHMs should not be confused with LLMs, which take unstructured textual inputs (e.g., clinical notes or medical histories). That said, LLMs are also finding increasing application in longevity research^56^; for instance, it has recently been shown that an LLM-derived biological age outperforms epigenetic clocks in predictive accuracy of multiple chronic age-related diseases as well as all cause-mortality^57^.

LHMs will arguably become more beneficial for practical longevity research than the much-debated aging clocks. Already, they inherently encompass the properties required of aging clocks and mortality predictors, at least regarding health assessment. Yet, their utility for deepening our understanding of aging—like that of aging clocks—remains to be shown. Additionally, it is straightforward to derive an analog of biological age through a relatively simple postprocessing procedure applied to LHM outputs, similar to what is done for second-generation clocks. For example, it can be done by finding an age that maximizes the likelihood of an observed sequence of health events for a given individual. The recently proposed LHMs, including BEHRT, Life2Vec, and Delphi-2M, clearly demonstrate how the access to vast amounts of longitudinal data enables deep insights and accurate predictions of individuals’ health and even their socioeconomic status^53–55^. Given the wide scope of problems LHMs promise to solve—provided that sufficiently large and high-quality datasets are available—the previous three approaches may appear comparatively limited; however, in data-scarce settings, we must still rely on these classic instruments.

## Aging clocks meet aging theories

The geroscience hypothesis posits that aging itself acts as a system-level risk factor that increases vulnerability to chronic conditions across organs and tissues^26,58^. Within this view, a global estimate of aging is justified if it (i) aggregates information across tissues, (ii) predicts clinically relevant outcomes better than chronological age, and (iii) guides actionable prevention or intervention strategies. Our discussion is therefore not directed at the geroscience hypothesis per se, but at the current implementations of biological age proxies, which often lack clear definitions, uncertainty estimates, and within-domain validation.

Exploring the theoretical value of aging clocks, we must further ask whether they bring us any closer to understanding the fundamental nature of aging. Clearly, the quest for primary biomarkers of aging remains important, regardless of the criticisms towards the concept of biological age. Yet, identifying such markers—especially those reflecting the earliest, hard to detect signs of aging—requires a much deeper understanding of the aging process. The ML approaches currently used to construct aging clocks are not designed to address the root causes of aging, as they focus on learning correlations rather than causal relationships: they are not trained to distinguish between passengers and drivers of aging^59^. This is clearly underscored by the fact that numerous accurate first-generation epigenetic clocks could be constructed using virtually non-overlapping combinations of DNA methylation sites^60^ and by other evidence^52^. There have been several attempts at drawing an aging theory centered around epigenetic clocks^61–63^, but more than a decade after their introduction, the exact molecular pathways and actionable targets are yet to be discovered. At present, training ML models still appears insufficient to reveal what constitutes a true primary aging biomarker. Consistent with this, we view prediction and mechanistic interpretation as complementary goals: predictive models can prioritize hypotheses and track responses, but causal claims require targeted designs and perturbation testing.

The hallmarks of aging^64^ represent an effort to dissect aging by cataloging its manifestations, which yielded an unweighted, undirected graph that treats all elements as equally important, without integrating them into a coherent theory. This framework portrays aging as an agnostic phenomenon, described empirically but lacking explanatory depth. The proliferation of aging clocks based on diverse and often interchangeable (redundant?) biomarkers reinforces this perception. Even reaching a consensus on the “perfect” predictor of biological age would hardly guarantee to clarify the fundamental causes of aging, as such consensus would presently be built on correlational patterns rather than causal understanding.

All the more interesting and timely, therefore, are attempts to develop aging clocks grounded in theory-driven assumptions—especially given that most current clocks are seldom used to inform or refine theories of aging. Notable recent exceptions include PRC2-based clocks^65^, transposable element-based clocks^66^, and stochasticity-based clocks^67^. However, the features and coefficients of most clocks remain difficult to interpret, and mechanistic or actionable insights derived from them are extremely scarce, with only a few recent preprints offering promising leads, such as those investigating cellular rejuvenation without loss of somatic identity^68,69^. In contrast, future efforts could prioritize a more theory-informed approach: selecting clock features based on clear, biologically grounded hypotheses, employing or developing rigorous frameworks to screen for actionable and experimentally verifiable targets.

It is also reasonable to expect that future integration of established biological principles into causal inference approaches^70^ could help address some challenges in selecting biomarkers that reflect the drivers of aging. Intriguingly, an attempt has already been made to identify potentially causal CpG sites using the Mendelian randomization-based approach^71^. However, even causal inference may be insufficient to prove the robustness of a given biomarker without a classical laboratory experiment involving its perturbation followed by survival curve or other relevant analyses^72^.

Therefore, deciding which specific biomarkers to include in longevity intervention studies remains a complex and debated question. A well-grounded theory of aging would potentially narrow the list, but until such a framework emerges, the more comprehensive the biomarker panel is, the better. Validating any longevity or rejuvenation therapy will inevitably require an extensive suite of functional and biochemical investigations; a composite health index alone is not supposed to benchmark interventions.

## The simplification effect and two paradigms of biological age estimation

Despite the shortcomings discussed above, there still seems to be a viewpoint from which biological age can appear useful, nicely illustrated by the following passage: *"Typically, feature attributions can be difficult to understand for non-machine-learning practitioners because they are usually in units of predicted probability or logits units. To make our biological age explanations more accessible, we rescaled our attributions to the age scale in units of years…"*^73^. Hence, biological age can serve as a convenient, intuitively simple measure that compresses complex statistical concepts for non-specialists. While making intricate technical ideas more accessible is indeed appealing and helpful, we must ask whether striving for a better estimation of this measure—rather than improving tools for direct health outcome prediction—is truly valuable and worthwhile.

Within this view, biological age helps non-specialists navigate statistical deep waters, and the efforts to develop aging clocks take on a new light. Each new generation of aging clocks, pursuing a better estimate of biological age, encounters new challenges. For example, first-generation aging clocks^5,11^ (Fig. 1c) interpret the model-predicted chronological age as directly equivalent to biological age, and the error a model makes when predicting chronological age is interpreted as age acceleration. As discussed above, this interpretation suffers from the biomarker paradox. Second-generation clocks (Fig. 1d), such as PhenoAge^6^ or GrimAge ^7^, essentially represent a classic ML pipeline where the target outcome is all-cause mortality (and biological age is calculated from the predicted partial hazards). Particularly, this formulation itself explicitly aims to satisfy the third property of the definition of biological age, and—as downstream analyses show—the resulting aging clocks also confidently predict the risk of age-related diseases^8,46^ and effectively discriminate between diseased and healthy individuals^3^.

We can expect that in the near future, aging clocks will emerge that are trained simultaneously to distinguish risks of all-cause mortality and multimorbidity, thereby fully aligning with our definition. Notably, such clocks are in fact designed to predict biological age based on risks of aging-related diseases, which is fully consistent with the definition of an aging biomarker proposed by Mikhail Blagosklonny^74^: “*…the sum of all age-related diseases is the best biomarker of aging…*”. If we consider that the risk of each age-related disease (health outcome) can be predicted from biomarkers using either machine learning models or expert assessment, then we can formulate a mathematical definition of biological age as the age that maximizes the likelihood of observing the distribution of risks predicted by the best available predictive approaches (analogous to how biological age is estimated in second-generation aging clocks). This definition is fully consistent with the geroscience hypothesis, since simultaneously reducing the risks of multiple age-related diseases would inevitably lead to increased lifespan.

**Fig. 2:**
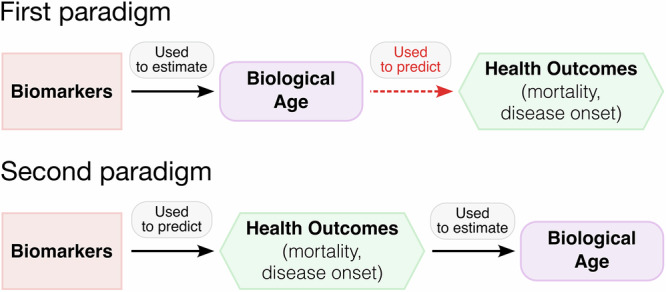
Two paradigms of biological age estimation. In the first paradigm, biomarker data are compressed into a single latent quantity representing either the current state (e.g., first-generation epigenetic clocks) or the rate (e.g., DunedinPACE) of biological aging. Examples include first-generation epigenetic clocks, the Klemera-Doubal model, PCA clocks, and DunedinPACE. The red dashed arrow illustrates the idea that quantities within the first paradigm are not specifically designed for health outcome prediction—yet this is typically expected of them. In the second paradigm, biomarkers are used to train an ensemble of models that directly predict risks of age-related diseases—including all-cause mortality—with these risks then aggregated into a unified biological age estimate. Examples include second-generation epigenetic clocks; additionally, survival ML models, expert-derived risk scores, and Large Health Models (LHMs) fall into this category if paired with a downstream biological age calculation step.

Thus, we formulate two paradigms for predicting the biological age (Fig. 2). In the first paradigm, models compress information from biomarkers (e.g., methylation levels at hundreds of CpGs) into a single latent quantity aimed at estimating either the biological age itself or its rate of change (e.g., DunedinPACE). The examples include first-generation epigenetic aging clocks^5^, the Klemera-Doubal approach^45^, PCA-based clocks^75^, and DunedinPACE^48^. In the second paradigm, one first develops a model or an ensemble of models that predict risks of age-related diseases, including all-cause mortality. These predicted risks are then used to compute biological age. Examples currently include second-generation epigenetic^6,7^ and clinical^73^ clocks, but could be updated with survival ML models, disease-specific expert assessments, and LHMs predicting biological age via a downstream procedure. The advantage of the second paradigm is that it can simultaneously ensure accuracy in predicting diverse health outcomes and generalizability, by aggregating risk estimates into a unified biological age. Its obvious drawback, however, is the requirement for large volumes of longitudinal high-quality data needed for training.

## Uncertainty estimation and limits of applicability

**Fig. 3:**
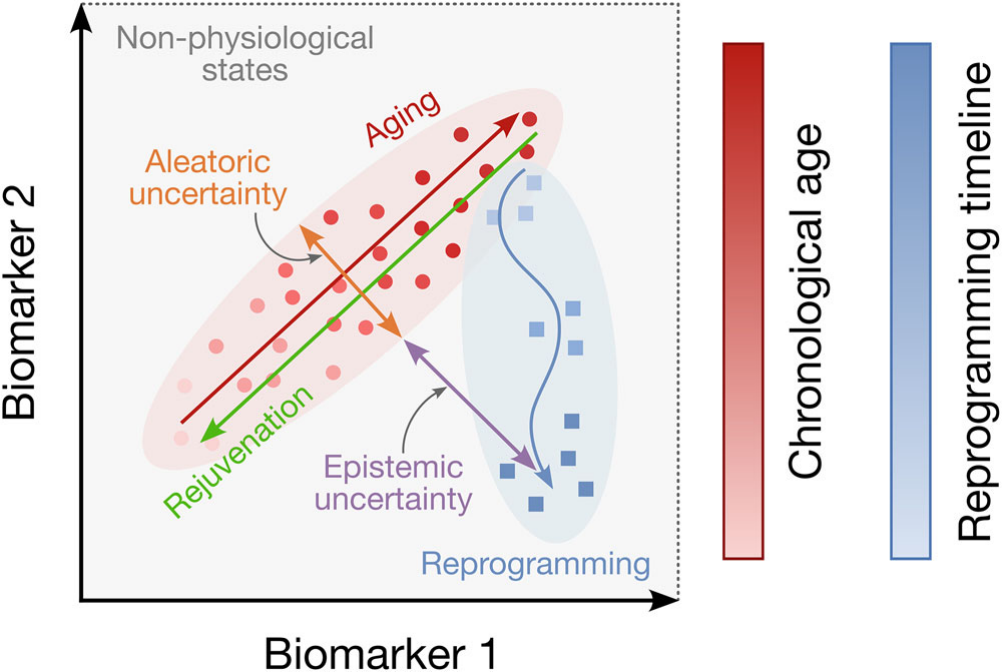
Shift in data distribution creates uncertainty of biological age estimation. The data samples (e.g., patients or cells) are represented as points in the biomarker space (two-dimensional in the demonstrated case: e.g., defined by two CpG methylation sites or two principal components). With aging, these points move along a characteristic aging trajectory (red arrow), whereas true rejuvenation would correspond to a movement in the opposite direction (green arrow). When a model is trained on noisy aging samples, it learns the general trend of biomarker changes associated with aging, but its predictive performance is inherently limited by data noise—referred to as aleatoric uncertainty—and is typically quantified using in-distribution metrics such as mean absolute error (MAE). However, when the model is applied to data outside the training distribution, it becomes subject to additional epistemic uncertainty, which MAE does not capture. This occurs, for example, when aging clocks trained on tissue samples from normally aging individuals are applied to data from in vitro reprogrammed cells (blue arrow). Although the projection of the reprogramming trajectory onto the aging axis may appear as rejuvenation, the actual state of reprogrammed cells no longer lies within the distribution of physiological states observed in the training data.

Finally, a problem common to all generations of aging clocks, yet consistently overlooked, is the evaluation of prediction uncertainty^20,76^. Nearly all published aging clocks provide point estimates without confidence intervals, in drastic contrast to classical diagnostic instruments such as glucometers or blood pressure monitors, which report measurement errors in their user manuals. Importantly, metrics such as mean absolute error (MAE) cannot serve as such confidence intervals because they only capture how the model performs on data similar to what it was trained on (known as aleatoric uncertainty, Fig. 3). However, they cannot account for the additional uncertainty that arises when the model encounters data that differs from its training set—such as samples from different countries, ethnicities, tissues, or other experimental conditions (epistemic uncertainty, Fig. 3)^20,76^. The lack of uncertainty estimation has already led to misleading interpretations when epigenetic clocks trained on healthy tissues were applied to assess the age of cells reprogrammed in vitro^20^. Any significant differences between the datasets used to train the clocks and those to which they are applied introduce uncertainty that must be accounted for when estimating biological age (Fig. 3).

In other words, it is important to “stay within the domain” when applying aging clocks to a new dataset. One could object that such a requirement excludes the possibility of applying aging clocks trained on human blood samples to cell cultures during cellular reprogramming to test rejuvenation effect of this genetic intervention, to measure biological age trajectories during embryogenesis^77^, or to apply clocks trained on one species to another. This, in turn, limits the potential to use established aging clocks for assessing the effects of putative rejuvenation interventions in vitro. Indeed, the extrapolation of existing clocks on such experiments might be seen as a logical next step from overall human health assessment to cellular health assessment. However, moving down the biological scale of hierarchy (organism → tissue → cell) increases the risk of OOD predictions coming from the algorithms tuned for larger scales. At finer scales, using both frailty-like indices and biological clocks trained on samples from adult humans becomes less reliable and interpretable and is likely to yield spurious conclusions: therefore, we recommend avoiding such practices, especially in cases when per-sample prediction uncertainty is not quantified^20^. Instead, attention should be directed toward the cell-level alternatives which, although being less easily translatable to the level of organismal health, operate on explicit biological entities.

For example, while the frailty index is well-defined at the organismal scale for humans, it does not have direct analogs for a single cell or an in vitro cell culture. Rather than extrapolating frailty index or aging clocks on these samples, we can adopt scale-appropriate constructs. At the tissue or primary culture level (e.g., human fibroblasts), a composite cellular health panel can be defined from complementary, as non-redundant measurements as possible, spanning distinct functional axes (e.g., proliferation, DNA damage, mitochondrial function, stress resilience, viability, morphology, and chromatin/transcriptome state), and combined into a continuous score with documented uncertainty. At the single-cell level, one may quantify cellular health state from multi-modal features (e.g., transcriptomic senescence signatures, chromatin accessibility states, mitochondrial gene expression burden, DNA damage) and report per-cell uncertainty. For model organisms beyond humans (e.g., *C. elegans*), scale-appropriate phenotypes (locomotion/pumping rates, stress resistance, brood size dynamics, etc.) can be combined into organism-specific health indices. Across scales, we recommend: (i) to define the target state prospectively; (ii) to specify the measurement domain (assay, species, tissue); (iii) to use head-to-head benchmarks against relevant outcomes (e.g., survival, stress tolerance, functional decline); and (iv) to report domain limits and per-sample uncertainty. This extends the concept of health state indices to the cellular level and prevents the out-of-domain overreach.

As a brief recommendation, based on the reasoning above, we propose the following simple rule to define the limits of applicability of aging clocks: “Stay within the domain the model was trained on”. In other words, if a clock was trained on blood samples from adult humans, for example, then it should be applied only to blood samples from adult humans. Thus, aging clocks remain useful tools within their validated domains, but their application beyond those contexts requires caution and explicit verification that the target data share the same underlying distribution and assumptions as the training set.

In summary, an incorrect interpretation of biological age—especially when prediction uncertainty is ignored—can lead to excessive anxiety or, conversely, unwarranted reassurance about one’s health or clinical outcomes. All mentioned concerns make biological aging clocks a potentially risky tool in their current form, highlighting the need for meticulous research and elaborate discussion about their concept and limitations.

## Conclusion and outlook

Longevity medicine is advancing rapidly, emphasizing the need for consensus on biomarkers that truly capture biological aging, not only in individuals with age-related conditions, but also proactively in healthy individuals. Equally important is the development of predictive models that can link these biomarkers to clinically relevant outcomes. In this Perspective, we have discussed whether aging clocks contribute meaningfully to these objectives.

Given the challenges surrounding current aging clocks and the availability of alternatives to assess human health, we summarize their limitations and propose possible solutions for the three primary goals of aging clock research (Table 1).

**Table 1:** Revisiting the goals for constructing aging clocks

Goal	Current clock problems	Possible solutions/Suggestions
Predicting clinical trial endpoints	• There are well-established, explicit surrogate endpoints in clinical trials; **aging clocks lack comparable validation**	• Conduct **extensive, open-source benchmarking** of aging clocks against **well-defined criteria** targeting adverse health outcomes
	• **ML models already predict** clinical outcomes **directly** instead of biological age	• Adopt or develop **radically more advanced methodologies** for aging clock training
	• Emerging **large health models** promise to overtake aging clocks, as they are supposed to inherently capture their properties of outcome predictor and health state estimator	• **Integrate or replace clocks** with established or emerging methodologies for health outcome prediction
Better understanding of the aging process	• Clock training is rarely based on theory-driven assumptions, and is hence rarely used to inform any theory of aging (notable exceptions: PRC2 clocks^65^, transposable elements clocks^66^, and stochasticity-based clocks^67^)	• Focus on clear, **theory-driven assumptions** behind selecting particular features before training aging clocks
	• Clock features and coefficients are usually **difficult to interpret**	• Employ or build rigorous clock-based frameworks to **screen for actionable and verifiable targets**
	• Mechanistic and actionable insights derived from aging clocks are extremely scarce (a short list of exceptions includes preprints investigating factors rejuvenating cells without loss of somatic identity^68,69^)	• Develop clocks **for chronological** (“forensic”) age prediction, which is, by itself, of certain scientific interest
Simplified estimate of “overall health”	• There are **simpler, better interpretable measures** (e.g., frailty index for older individuals, intrinsic capacity score and other geroscience-aligned multi-system composites, disease risk scores, etc.)	• **Acknowledge** that biological age merely serves as a **convenient measure** that simplifies and compresses complex statistical concepts **for non-specialists**
	• Reducing health to a single variable could **oversimplify the results** of exploratory studies	• **Fully clarify the challenges and uncertainties** of aging clock predictions, especially when applying them in non-scientific contexts, including personal health assessment
	• Challenges related to clock algorithms and training data quality make the clocks **unreliable for personal predictions**, outside of population-level studies	• Improve clock algorithms to **provide explicit estimation of uncertainty (confidence intervals)** for every biological age prediction, in addition to MAE
	• Most published clocks **cannot report the uncertainty of their predictions**, making them even more prone to misinterpretation and unsafe use	

In summary, we conclude that all limitations of aging clocks are hypothetically solvable. Whether solving them is worth the effort, that is a more difficult question. From the perspective of the geroscience hypothesis, investing resources in tools that can directly predict age-related risks of death and disease would be a perfectly valid approach. ML algorithms evolved to replace expert-based scores in such tasks, and the emerging LHMs promise to perform even better and tackle an even wider variety of related issues, although their superiority remains to be shown, and their construction relies heavily on data abundance.

So, *do we actually need aging clocks?*

If our goal is to develop a surrogate endpoint for clinical trials of geroprotectors or to construct an intuitive measure that reflects an individual’s overall health status, then the answer is probably yes: but only if these clocks are accurate, consistent, generalizable, and provide explicit estimation of prediction uncertainty—a level of performance achievable through rigorous and extensive validation, and through developing novel methods for clock construction and uncertainty estimation.

We believe the most effective path toward such clocks aligns best with shifting the logic of clock construction—from the first paradigm, where the biological age serves as a compact summary of biomarkers calibrated to the chronological age, to the second paradigm, where the biological age is redefined as a single, interpretable number that encapsulates risks of multiple age-related diseases. This approach requires huge datasets to predict the risks of death and disease, but its practical implementation would ultimately yield the most reliable estimate of the biological age. In other words, aging clocks could serve as a meta-layer built atop an ensemble of ML models predicting diverse age-related disease risks or, alternatively, as a result of post-processing outputs from LHMs.

On the other hand, if our goal is to understand the biology of aging, then the answer is less clear, although emerging examples show that certain aging clocks could aid in finding novel gene targets or pathway regulators. That said, we suggest considering the integration of existing and novel causal inference frameworks, and bold, theory-driven assumptions into aging clocks (provided that the uncertainty and other clock issues are controlled for)—approaches that have the potential to yield truly unexpected and transformative insights.

Science evolves in diverse ways, and each study yields its own answer to the question of how useful the aging clocks are. Current aging research, rich in ML-based techniques, offers a unique opportunity to explore new methods and theories of aging that can complement and expand our knowledge, perhaps beyond aging clocks. We hope that, through transparent reasoning, the emerging longevity medicine informed by longevity science will push further the boundaries of healthy lifespan.

## Data Availability

No datasets were generated or analyzed during the current study.
